# Hemichorea-hemiballismus as an initial manifestation in a Moroccan patient with acquired immunodeficiency syndrome and toxoplasma infection: a case report and review of the literature

**DOI:** 10.4314/pamj.v10i0.72216

**Published:** 2011-09-25

**Authors:** Samira Rabhi, Kawthar Amrani, Mustapha Maaroufi, Zineb Khammar, Hajar Khibri, Maha Ouazzani, Rhizlane Berrady, Siham Tizniti, Ouafae Messouak, Faouzy Belahsen, Wafaa Bono

**Affiliations:** 1 Department of Internal medicine, Hassan II University Hospital, Faculty of Medicine and Pharmacy, University Sidi Mohammed Ben Abdellah, Morocco; 2 Department of Radiology, Hassan II University Hospital, Faculty of Medicine and Pharmacy, University Sidi Mohammed Ben Abdellah, Morocco; 3 Department of Neurology, Hassan II University Hospital, Faculty of Medicine and Pharmacy, University Sidi Mohammed Ben Abdellah, Morocco

**Keywords:** Hemichorea ballismus, acquired immunodeficiency syndrome, HIV, cerebral toxoplasmosis

## Abstract

Neurologic signs and symptoms may represent the initial presentation of AIDS in 10-30% of patients. Movement disorders may be the result of direct central nervous system infection by human immunodeficiency virus (HIV) or the result of opportunistic infections. We report the case of a 59 years old woman who had hemichorea-hemiballismus subsequently found to be secondary to a cerebral toxoplasmosis infection revealing HIV infection. Movement disorders, headache and nausea were resolved after two weeks of antitoxoplasmic treatment. Brain MRI control showed a marked resolution of cerebral lesion. Occurrence of hemichorea-ballismus in patient without familial history of movement disorders suggests a diagnosis of AIDS and in particular the diagnosis of secondary cerebral toxoplasmosis. Early recognition is important since it is a treatable entity.

## Introduction

Berger et al. in 1984 [1] were the first to report involuntary movements in AIDS on the literature. This was followed by the descriptions of Navia et al. in 1986 [2] and Nath et al. in 1987 [3]. Since then, a wide variety of movement disorders have been described, including: Parkinsonism, hemichorea-ballismus, myoclonus, dystonia, tremor, and painful legs and moving toes [4]. Hemichorea–ballism is usually an acute complication in patients already diagnosed with AIDS. Some studies have reported them as the second most frequent movement disorder after Parkinsonism [5]. However, in a few instances this movement disorder may be the first symptom of HIV infection [6]. Ballism most commonly has been attributed to lesions of the subthalamic nucleus (STN), however, it may result from a variety of other lesions. A subthalamic toxoplasmic abscess is the most common cause of hemichorea-ballism in AIDS patients. However, the presence of hemichorea–ballism in patients with cerebral toxoplasmosis is not common [7]. The management of patients with HIV-related hemichorea–ballism includes the diagnosis and treatment of opportunistic infections and the use of HAART. Although other authors found that clinical response to antitoxoplasmosis therapy was poor, with little or no improvement of the movement disorder [8]. We report a patient who showed hemichorea-ballism before diagnosis of AIDS with complete recovery by anti-toxoplasma therapy.

## Case report

The patient was a 59 year-old, heterosexual Moroccan woman who came to hospital with uncontrollable movements of her left arm and leg, progressed during four weeks. The involuntary movements gradually increased in frequency as the last week. She complained of a dull headache, intermittent nausea and vomiting. She denied a history of acquired immunodeficiency, rheumatic heart disease or using intravenous drugs. She had never been exposed to neuroleptics.

The neurological examination revealed continuous choreic movements of her left hand and foot and ballistic movements of her proximal left arm. The arm was more involved than the leg. The muscle tone was slightly decreased on the left compared with the right side. She was able to walk. Cranial nerve, sensory, cerebellar, and deep tendon reflex examinations were normal.

On physical examination, her blood pressure was 128/70 mm Hg; heart rate 90 beats/min; respiratory rate 16/min; and temperature 37,4°C. The general physical examination was unremarkable. Chest X-ray and electrocardiogram were normal. A computed tomographic (CT) scan of the brain on admission revealed a right capsulothalamic nodular enhancing lesion with surrounding edema.

**Figure 1:**
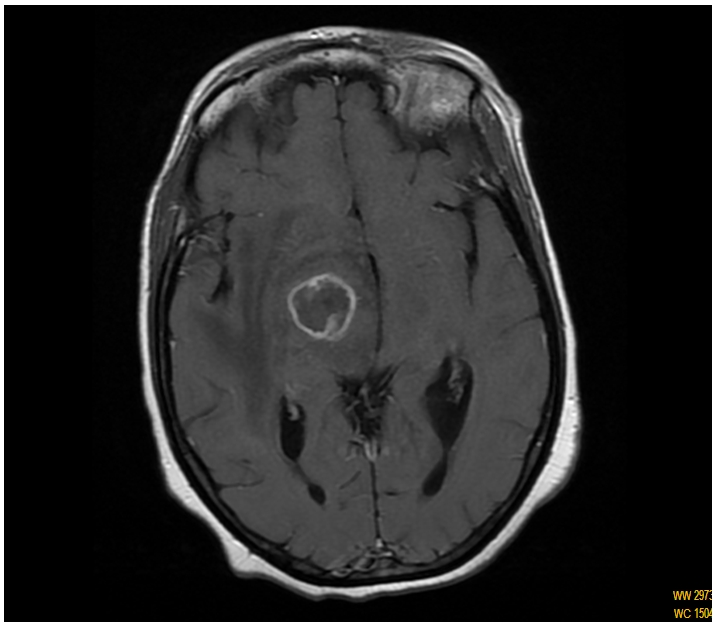
T1-weighted magnetic resonance imaging scan showing ring enhancing of the capsule-thalamic lesion after gadolinium injection in a Moroccan patient with hemichorea-hemiballismus as an initial manifestation of acquired immunodeficiency syndrome and toxoplasma infection

Laboratory findings included a white blood cell count of 5200/mm^3^, lymphopenia 500/mm^3^,, hemoglobin 12g/dl, platelets 229000/mm^3^,. She was found to be HIV positive by ELISA and Western blot analysis. CD4 cell level of 91/mm^3^, and Copy numbers of HIV-RNA were 718190 /ml. Titer of Toxoplasma gondii IgG was >250 UI/ml and specific IgM was positive. A serologic test for syphilis was negative. HBsAg was positive, HBe antibody and delta were negative, copy numbers of hepatitis B virus were 899000/ml and hepatitis C virus antibody was negative. Ten days again later, the patient developed a left hemiplegia with facial paralysis and confusion. Brain MRI showed a right capsulothalamic focal lesion characterized by a peripheral enhanced T2 hypointense and central hyperintense T2, appearance necrotic, with perilesional edema, hypointense on T1 and hyperintense on T2 and mild compression of third and lateral right ventricle suggesting toxoplasmic abscess (Figure 1).

Trimethoprim-sulfamethoxazole 50mg/kg/day (TMP-SMX) therapy was instituted associated with steroid. This treatment was started immediately after result of brain CT scan. Pyrimethamine was not available. She did not receive dopamine blocker. Movement disorders are resolved after two weeks since the start treatment and disappearance of headache and nausea. Brain MRI control showed marked resolution of cerebral lesion.

## Discussion

Involuntary movements in patients with AIDS have increasingly been recognized. Etiologic factors have included HIV encephalopathy, progressive multifocal leukoencephalopathy, Whipple's disease, and drug-induced movement disorders [2,3]. However, one of the most frequently reported cause of involuntary movements in patients with AIDS is toxoplasmosis [7]. Toxoplasmosis is one of the most common opportunistic brain infections in AIDS, accounting for ≤30% at postmortem studies, particularly when CD4 levels are <300/mm^3^ [8]. Cerebral toxoplasmosis most often presents as focal neurological signs of subacute onset. A number of movement disorders have been described in this patient population. Most commonly described is a unilateral hyperkinetic disorder. Reports of Parkinsonism, dystonia, and akathisia have also been described. Hemichorea–hemiballism in patients with cerebral toxoplasmosis is not common, occurring in only 7.4% of cases [9]. In Piccolo et al.'s series [6] of 51 sporadic cases of chorea, 5/51 patients had chorea in association with AIDS. Two patients had chorea secondary to toxoplasmosis, with one having a toxoplasma abscess in the contralateral STN and one having basal ganglia toxoplasmosis. Piccolo et al. concluded that AIDS-related disease should be considered in young patients presenting with chorea without a family history of movement disorders.

Cerebral toxoplasmosis precipitated these movement disorders is highly suggestive due to the sudden onset of the movement disorder associated with the appearance of a toxoplasma abscess in the appropriate anatomical location and can unmask movement disorders in patients with HIV-1 infection [3]. The basal ganglia are involved very early in the course of HIV-1 infection. Autopsy studies confirm that the basal ganglia is invariably infiltrated by HIV infected microglia and multinucleated giant cells [2,10]. Our case was characterized by continuous choreic movements of her left hand and foot and ballistic movements of her proximal left arm. The clinical picture of AIDS related hemichorea–ballismus does not differ from that in patients without HIV infection. The hyperkinetic movements may affect proximal and/or distal muscles of the extremities and range from ballistic to choreic or choreoathetotic movements. Hemichorea-ballism and even generalized chorea may be the initial presenting symptom of AIDS [5,7].

Ballism was defined by Meyers as “more or less extensive, vigorous, rapidly executed, poorly patterned nonadaptive and seemingly purposeless activities of appendicular, truncal and/or faciocephalic striated muscles”. The involvement of proximal limbs including large rotatory excursions has been stressed [11]. Conceptual and nosological controversies exist on the differentiation of ballism from chorea. While quantitative and qualitative criteria are used to define the phenomenological entities, clinical observations and experimental data suggest that chorea and ballism are parts of a continuum of movement disorders [3,12].

The majority of cases of chorea-ballism in AIDS reported multiple cerebral lesions rather than a single one. Tumor-like lesions frequently provoke intracranial hypertension, confusional state, and/or focal signs mainly of the pyramidal type. Movement disorders are seldom observed when these kinds of lesions occur; when they do occur, their most frequent form is hemichorea-ballism or hemichorea-athetosis [10]. The cerebral structures more commonly affected are the subthalamic nucleus, thalamus, and head of the caudate, putamen, globus pallidus, midbrain and internal capsule [7,13]. Ballism or chorea were convincingly associated with damage to the subthalamic nucleus or its efferent pathways, which removes excitation of the globus pallidus, thus disinhibiting the ventrolateral and ventroanterior thalamic nuclei receiving pallidal projections [14].

In our patient, the diagnosis of toxoplasmosis was presumptive, based on the presence of elevated IgG titers, imaging findings, and remarkable response to specific treatment, as no biopsy was performed. She had two unusual features: a toxoplasma abscess induced hemichorea-ballism, and a capsule-thalamic lesion was responsible for the movement disorder, revealing AIDS disease.

Movement disorders in our patient are resolved after two weeks since the start treatment and disappearance of headache and nausea with marked resolution of cerebral lesion in brain MRI control. The management of patients with HIV-related hemichorea–ballism includes the diagnosis and treatment of opportunistic infections, the symptomatic treatment of the movement disorder and the use of HAART [8]. Although other authors found that clinical response to antitoxoplasmosis therapy was poor, with little or no improvement of the movement disorder. The apparent lack of response of the movement disorder to antitoxoplasmosis therapy despite resolution of the abscesses could be due to persistent gliosis. Alternatively, the underlying HIV-1 infection may play a role in the persistence of the movement disorder [5,6,15].

## Conclusion

Cerebral toxoplasmosis is the most frequent opportunistic infection affecting the brain in patients with human immunodeficiency virus type 1 (HIV-I) infection and may be the initial presentation of acquired immune deficiency syndrome. Early recognition is important since it is a treatable entity.
